# Covered versus Uncovered Self-Expandable Metal Stents for Managing Malignant Distal Biliary Obstruction: A Meta-Analysis

**DOI:** 10.1371/journal.pone.0149066

**Published:** 2016-02-09

**Authors:** Jinjin Li, Tong Li, Ping Sun, Qihong Yu, Kun Wang, Weilong Chang, Zifang Song, Qichang Zheng

**Affiliations:** 1 Department of Hepatobiliary Surgery, Union Hospital, Tongji Medical College, Huazhong University of Science and Technology, Wuhan, China; 2 Department of Gastrointestinal Surgery, Union hospital, Tongji Medical College, Huazhong University of Science and Technology, Wuhan, China; University Hospital Llandough, UNITED KINGDOM

## Abstract

**Aim:**

To compare the efficacy of using covered self-expandable metal stents (CSEMSs) and uncovered self-expandable metal stents (UCSEMSs) to treat objective jaundice caused by an unresectable malignant tumor.

**Methods:**

We performed a comprehensive electronic search from 1980 to May 2015. All randomized controlled trials comparing the use of CSEMSs and UCSEMSs to treat malignant distal biliary obstruction were included.

**Results:**

The analysis included 1417 patients enrolled in 14 trials. We did not detect significant differences between the UCSEMS group and the CSEMS group in terms of cumulative stent patency (hazard ratio (HR) 0.93, 95% confidence interval (CI) 0.19–4.53; p = 0.93, I^2^ = 0%), patient survival (HR 0.77, 95% CI 0.05–10.87; p = 0.85, I^2^ = 0%), overall stent dysfunction (relative ratio (RR) 0.85, M-H, random, 95% CI 0.57–1.25; p = 0.83, I^2^ = 63%), the overall complication rate (RR 1.26, M-H, fixed, 95% CI 0.94–1.68; p = 0.12, I^2^ = 0%) or the change in serum bilirubin (weighted mean difference (WMD) -0.13, IV fixed, 95% CI 0.56–0.3; p = 0.55, I^2^ = 0%). However, we did detect a significant difference in the main causes of stent dysfunction between the two groups. In particular, the CSEMS group exhibited a lower rate of tumor ingrowth (RR 0.25, M-H, random, 95% CI 0.12–0.52; p = 0.002, I^2^ = 40%) but a higher rate of tumor overgrowth (RR 1.76, M-H, fixed, 95% CI 1.03–3.02; p = 0.04, I^2^ = 0%). Patients with CSEMSs also exhibited a higher migration rate (RR 9.33, M-H, fixed, 95% CI 2.54–34.24; p = 0.008, I^2^ = 0%) and a higher rate of sludge formation (RR 2.47, M-H, fixed, 95% CI 1.36–4.50; p = 0.003, I^2^ = 0%).

**Conclusions:**

Our meta-analysis indicates that there is no significant difference in primary stent patency and stent dysfunction between CSEMSs and UCSEMSs during the period from primary stent insertion to primary stent dysfunction or patient death. However, when taking further management for occluded stents into consideration, CSEMSs is a better choice for patients with malignant biliary obstruction due to their removability.

## Introduction

Most patients with a malignant biliary obstruction cannot undergo operations because of the advanced stage of the obstruction. Transhepatic or endoscopic stent insertion is the gold standard for palliative treatment to relieve biliary obstructions and improve quality of life [1–3]. Studies have shown that both plastic stent insertion and self-expandable metal stent (SEMS) insertion effectively relieve jaundice and improve quality of life [4, 5]. However, stent occlusion is the primary clinical problem associated with endoprostheses, leading to re-intervention [6]. Typically, plastic stents become occluded with sludge because of their small diameter. Enlarging the diameter seems to be a solution, but the diameter is limited by the endoscope delivery system [1, 7]. Uncovered SEMSs (UCSEMSs), which are only 3.5–4 mm in diameter, can expand to 8–10 mm in the bile duct after being released from the delivery system. Compared with plastic stents, UCSEMSs exhibit longer patency durations and lower occlusion rates, reduce the length of hospital stay and decrease the need for periodic stent exchange [8–10]. However, tumor ingrowth via the metal mesh has been observed in patients with UCSEMSs, which is one of the main causes of UCSEMS occlusion [11]. To prevent tumor ingrowth and avoid re-intervention, SEMSs covered with a non-porous membrane were developed.

Several trials have compared UCSEMSs and covered SEMSs (CSEMSs), but the conclusions have been inconsistent [12–19]. Moreover, two recent meta-analyses produced different conclusions [20, 21]. Thus, we performed a meta-analysis to reassess the efficacy of UCSEMSs compared with CSEMSs. This study specifically compares the cumulative stent patency rate, patient survival, stent dysfunction, overall complication rate and biliary drainage efficacy after CSEMS or UCSEMS placement to identify the more favorable option for palliative treatment of a distal malignant biliary obstruction.

## Methods

### Eligibility criteria

Types of studies: Randomized controlled trials (RCTs) comparing the efficacy of UCSEMSs and CSEMSs in treating distal malignant biliary obstruction were included. Non- and quasi-randomized trials were excluded.

Types of interventions: Both endoscopic and percutaneous approaches for stent placement for managing distal malignant biliary obstruction were considered. Both types of stents had to be commercially available and not handcrafted.

Types of participants: The study population was as follows: >18 years old, without gender restrictions, and diagnosed with obstructive jaundice caused by an unresectable tumor (not a hilar tumor).

All of the trials had been published in English.

### Search methods used to identify the studies

We conducted a comprehensive search of electronic databases (the EMBASE, PubMed, Science Citation Index Expanded and Cochrane Library databases) for trials from 1980 to May 2015. The search strategy was based on MeSH terms combined with key words. The detailed search strategies are described in S1 Table.

### Outcome choice

Cumulative stent patency and patient survival were considered as the primary outcomes. In this paper, only primary stent patency was analyzed. Primary stent patency is associated with an absence of recurrent symptomatic biliary obstruction and is equivalent to the duration from primary stent placement to stent dysfunction. Overall stent dysfunction, the overall complication rate and a change in serum bilirubin were secondary outcomes.

### Data collection

Two authors blinded to the authors and institutions of the search results selected the studies by following the preferred reporting items for systematic reviews and meta-analyses (PRISMA) process. Disagreements were solved through discussion with a third author. The reasons for exclusion were recorded. Two authors independently extracted the data from the included studies, and disagreements were discussed again with a third author.

The risk of bias in the included studies was assessed based on the recommendations in the Cochrane Handbook for Systematic Reviews of Interventions [22]. Two authors assessed the bias risk, and disagreements were discussed with a third author.

### Statistical analysis

Relative ratios (RRs) with 95% confidence intervals (CIs) were used to assess dichotomous data, and the weighted mean differences (WMDs) with 95% CIs were used to assess continuous data. We extracted hazard ratios (HRs) with 95% CIs from the publications as a relevant measure for the effects of stent patency and patient survival. We estimated the HRs from log-rank Chi^2^ statistics, log-rank p values, the given numbers of events, or Kaplan-Meier curves using the methods described by Tierney *et al* and Parmer *et al* [23, 24]. The Generic Inverse Variance method was applied to analyze time-to-event data.

We performed this meta-analysis following the guidelines in the Cochrane Handbook for Systematic Reviews of Interventions [22], and our report is in accordance with the PRISMA statement (S1 PRISMA Checklist). A fixed-effects model was used if no statistically significant heterogeneity was found; otherwise, a random-effects model was applied. Heterogeneities were analyzed by calculating the Chi^2^ and I^2^ statistics. I^2^>50% indicated substantial heterogeneity. If significant heterogeneity was found, the potential reasons for the heterogeneity were explored.

All statistical analyses were performed independently and in duplicate by two authors using RevMan 5.2 (Cochrane Collaboration, Oxford, UK) and Stata 11.0 (StataCorp, College Station, Texas, USA).

## Results

### Search results

**Fig 1:**
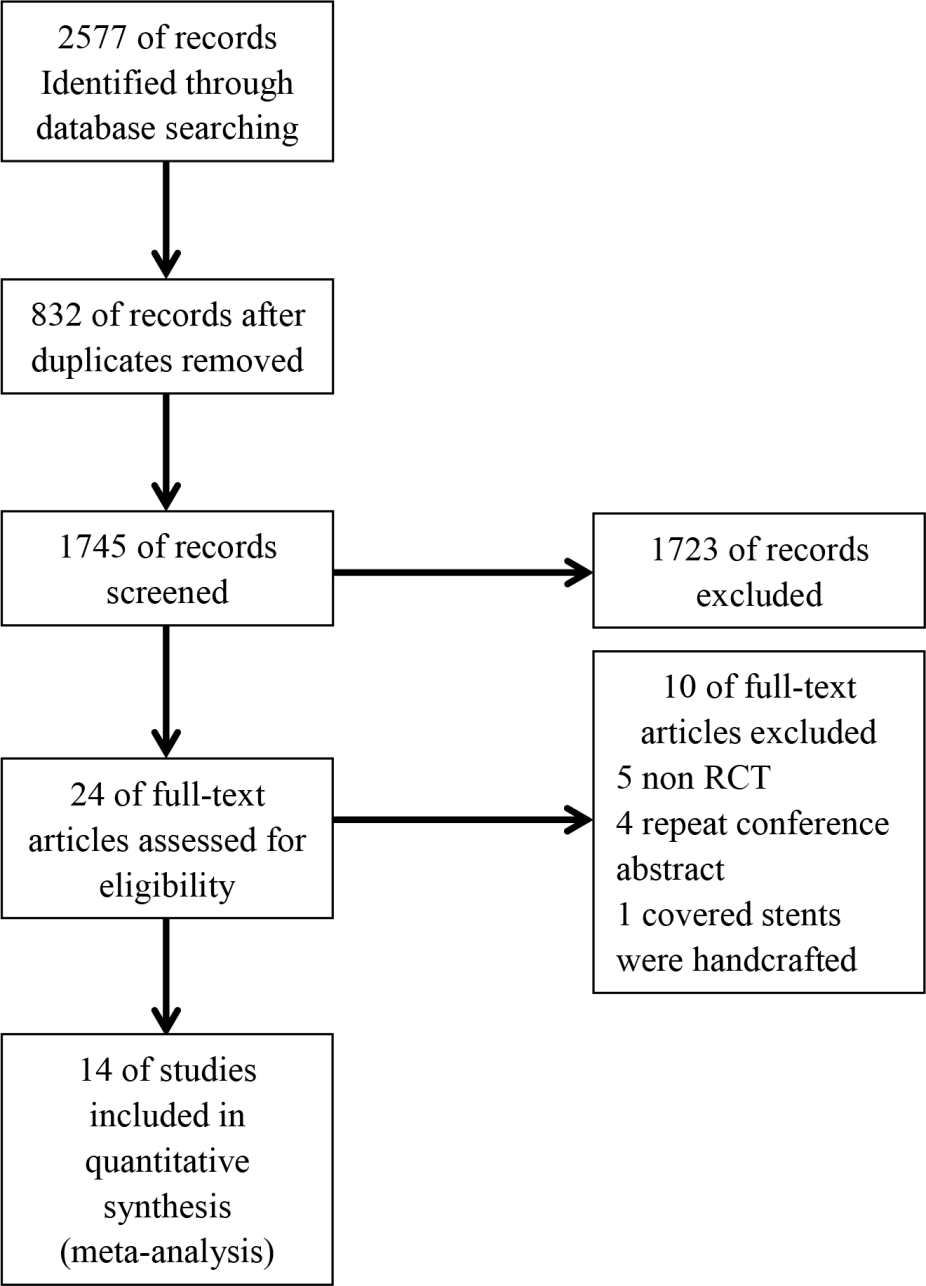
Study flow diagram.

The study screening process is shown in Fig 1. A total of 2579 potential abstracts and titles were identified through the systematic search. In all, 832 were duplicates, and a total of 1723 citations were excluded because they were not relevant to the studied topic. Among the remaining 24 citations, 5 trials were not RCTs, 4 were duplicate publications from a conference, and 1 involved handcrafted covered stents. Accordingly, we ultimately selected 14 trials for our meta-analysis.

A total of 1417 patients (700 with UCSEMSs and 717 with CSEMSs) were enrolled in the 7 full-text trials [12–16, 25, 26] and 7 abstract trials [18, 27–32] (Tables 1 and 2). Pancreatic cancer and bile duct carcinoma were the two main reasons for malignant biliary obstruction. Furthermore, 739 patients with pancreatic cancer and 156 patients with bile duct cancer were enrolled in 9 trials [12–16, 18, 25, 26, 31]. In 10 studies [12–14, 18, 26, 27, 29–32], stent placement was performed via an endoscope, and in 3 trials [15, 16, 25], the stents were inserted using a percutaneous approach. In the remaining trial, the method used to place the stents was unclear [28]. The patient characteristics (age and gender) at the baseline were similar between the two groups, and the dropout rates were not significant in any study.

**Table 1:** RCT studies included in meta-analysis.

study	stent	Covering materials	NO.of patients	Gender (M/F)	age(y)	stent patency (days)	patient survival (days)	No.of stent dysfunction	No. of complications
Kullman,E, 2010	uncovered		200	91/109	median 76	mean 154	Median 174	45	20
	covered	Polycarbonate- polyurethane	200	88/112	median 79	mean 199	median 116	47	14
Telford,J.J, 2010	uncovered		61	31/30	median 65	median 711	median 239	12	27
	covered	permalume	68	30/38	median 66	Median 357	Median 227	23	48
Kitano, M, 2013	uncovered		60	29/31	mean±sd 68.7±8.9	mean±sd 166.9±124.9	median 223	22	2
	covered	silicone	60	25/35	mean±sd 70.6±10.7	mean±sd 9.3±159.1	median 285	14	2
Krokidis,M, 2010	uncovered		30	16/14	mean 63.7	mean 166	median 180.5	9	4
	covered	ePTFE/FEP	30	20/10	mean 66.5	mean 227.3	median 243.5	4	3
Krokidis,M, 2011	uncovered		40	36/4	median 65	median 167	median 203.3	12	4
	covered	ePTFE/FEP	40	17/23	median 63.5	median 235	median 248	4	5
Lee,S.J, 2014	uncovered		20	9/11	mean±sd 63.2±11.7	mean±sd 413.3±63	mean 359	4	0
	covered	PTEF	20	9/11	mean±sd 62.1±8.6	mean±sd 207.5±46	mean 350	10	3
Ung, K.A, 2013	uncovered		34	9/25	median 79	median 127	median 157	NA	0
	covered	silicone	34	18/16	median 77	median 153	median 154	NA	2
Lee, S.H, 2004	uncovered		21	NA	NA	median 216	NA	11	NA
	covered	NA	22	NA	NA	median 127	NA	4	NA
Cho YD, 2009	uncovered		38	NA	NA	median 195	NA	NA	4
	covered	NA	39	NA	NA	median 227	NA	NA	10
Fukuda W, 2012	uncovered		71	NA	NA	median 314	NA	23	NA
	covered	NA	72	NA	NA	median552	NA	17	NA
Gonzalez-Huix F, 2008	uncovered		53	NA	NA	NA	NA	6	14
	covered	NA	61	NA	NA	NA	NA	15	23
Isayama H, 2000	uncovered		25	NA	NA	NA	NA	1	7
	covered	Polyurethane	25	NA	NA	NA	NA	4	1
Smits ME, 1995	uncovered		24	NA	NA	NA	NA	NA	3
	covered	Polyurethane	22	NA	NA	NA	NA	NA	3
Mangiavillano B, 2015	uncovered		21	NA	NA	Median 194	NA	NA	NA
	covered	NA	23	NA	NA	Median 89	NA	NA	NA

ePTFE/FEP: polytetrafluoroethylene and fluorinated ethylene propylene; PTEF: polytetrafluoroethylene

**Table 2:** Characteristics of RCTs in the meta-analysis.

Study	Stent length(mm)	Stent diameter (mm)	Approach of stent placement	Stent material	NO. of EST	Research center (s)	Country(ies)
Kullman,E, 2010	52/72	10	ERCP	alloy	200	multi	Sweden
Telford,J.J, 2010	NA	NA	ERCP	alloy	32	multi	America
Kitano, M, 2013	40/60/80	10	ERCP	alloy	60	multi	Japan
Krokidis,M, 2010	60–90	10/12	PTC	alloy	0	multi	Greece/Italy
Krokidis,M, 2011	40/60/80/100	10	PTC	alloy	0	multi	England/Italy
Lee,S.J, 2014	50–100	10	PTC	alloy	0	single	Korea
Ung, K.A, 2013	40/60/80	NA	ERCP	alloy	NA	multi	Sweden
Lee, S.H, 2004	NA	NA	ERCP	alloy	NA	single	Korea
Cho YD, 2009	NA	NA	ERCP	NA	NA	NA	Korea
Fukuda W,2012	NA	NA	NA	NA	NA	multi	Japan
Gonzalez-Huix F,2008	NA	NA	ERCP	alloy	NA	NA	NA
Isayama H,2000	NA	NA	ERCR	alloy	NA	multi	Japan
Smits ME,1995	NA	NA	ERCP	alloy	36	single	Netherlands
Mangiavillano B,2015	NA	NA	ERCP	alloy	NA	multi	Italy

EST: Endoscopic sphincterotomy; ERCP: Endoscopic Retrograde Cholangiopancreatography. PTC: Rercutaneous Transhepatic Cholangiography

### Risk of bias

We conducted a qualitative risk assessment for each trial. Of the 14 studies included in this meta-analysis, the risk of bias was considered unclear for the abstracts, whereas the full-text trials were assessed in accordance with the Cochrane Handbook for Systematic Reviews of Interventions (S1 and S2 Figs).

### Primary outcomes

#### Cumulative stent patency

**Fig 2:**
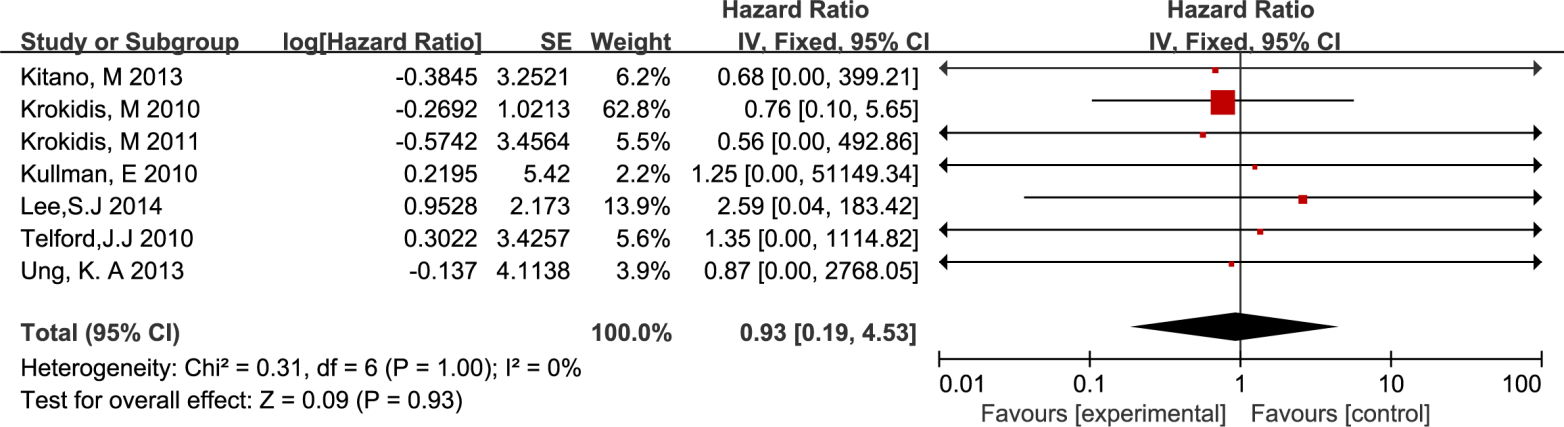
Forest plot of 7 trials addressing cumulative stent patency.

All included studies that were published as full-text articles compared stent patency between two groups. We did not detect a significant difference between the CSEMSs and the UCSEMSs (HR 0.93, IV, fixed, 95% CI 0.19–4.53; p = 0.93, I^2^ = 0%) (Fig 2).

#### Patient survival

**Fig 3:**
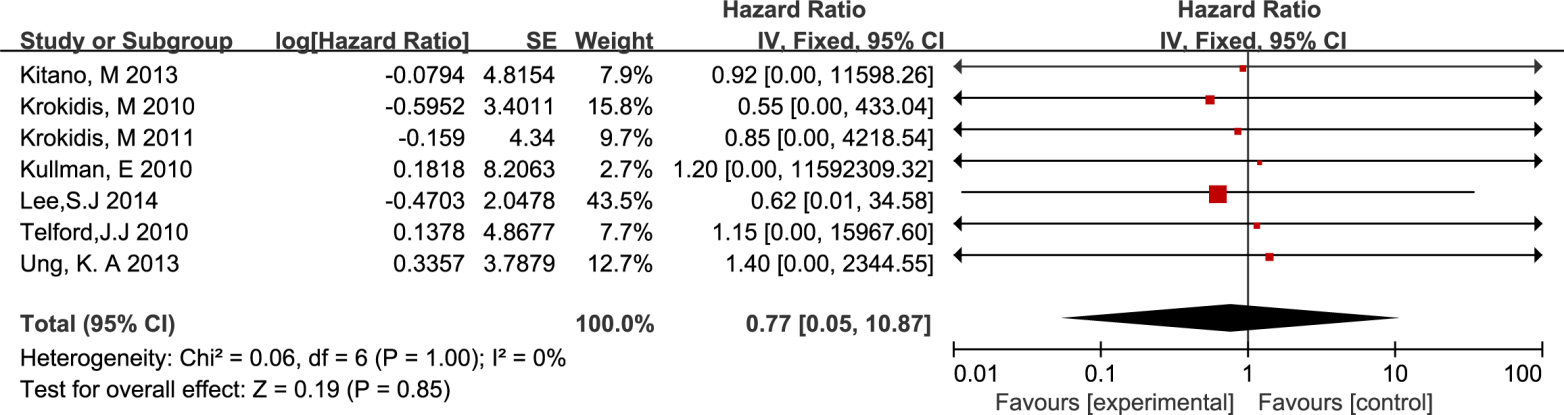
Forest plot of 7 trials investigating patient survival.

A summary of 7 full-text trials (Fig 3) revealed no statistically significant difference in overall survival between the two arms (HR 0.77, IV, fixed, 95% CI 0.05–10.87; p = 0.85, I^2^ = 0%).

### Secondary outcomes

#### Stent dysfunction

**Fig 4:**
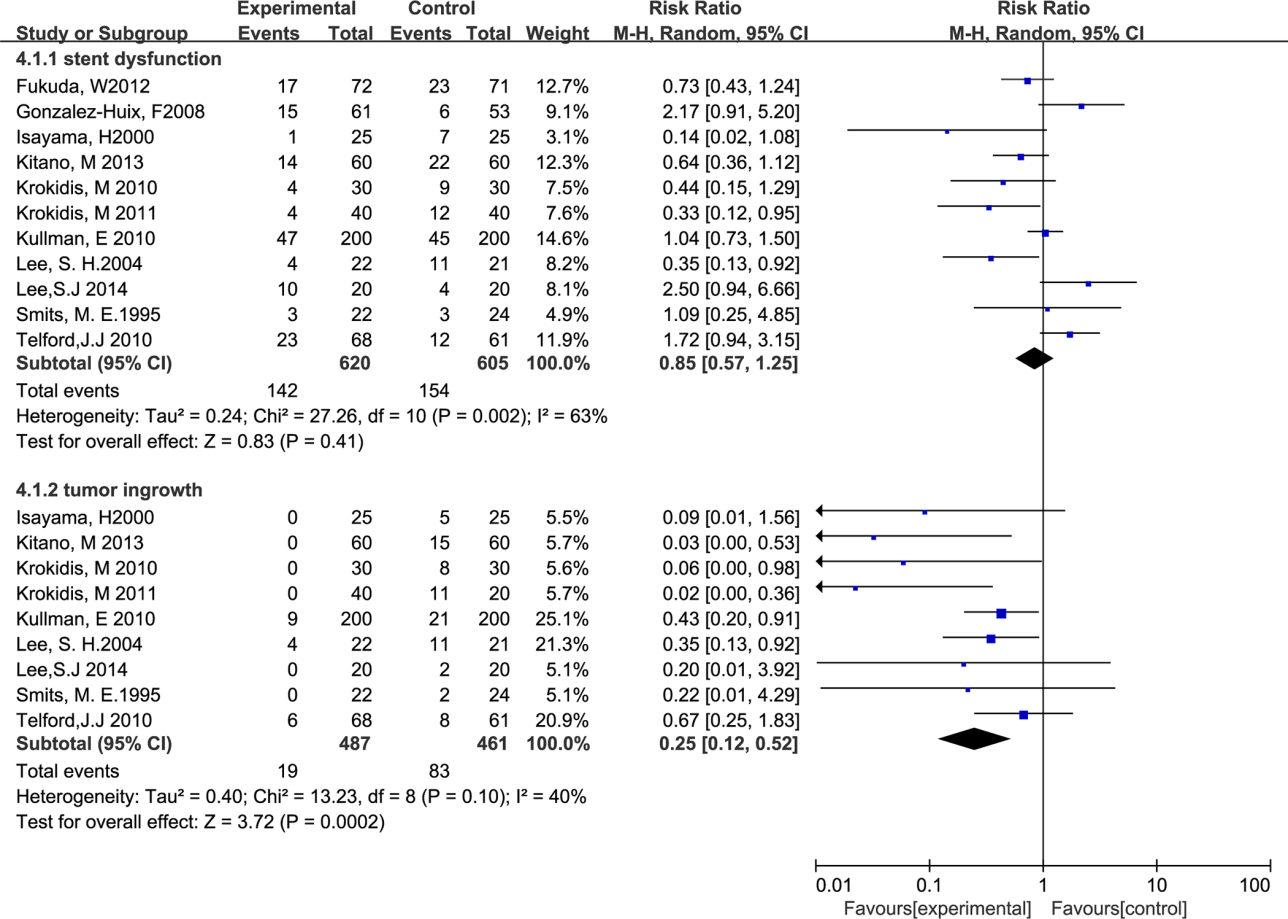
Forest plot of overall stent dysfunction and tumor ingrowth.

**Fig 5:**
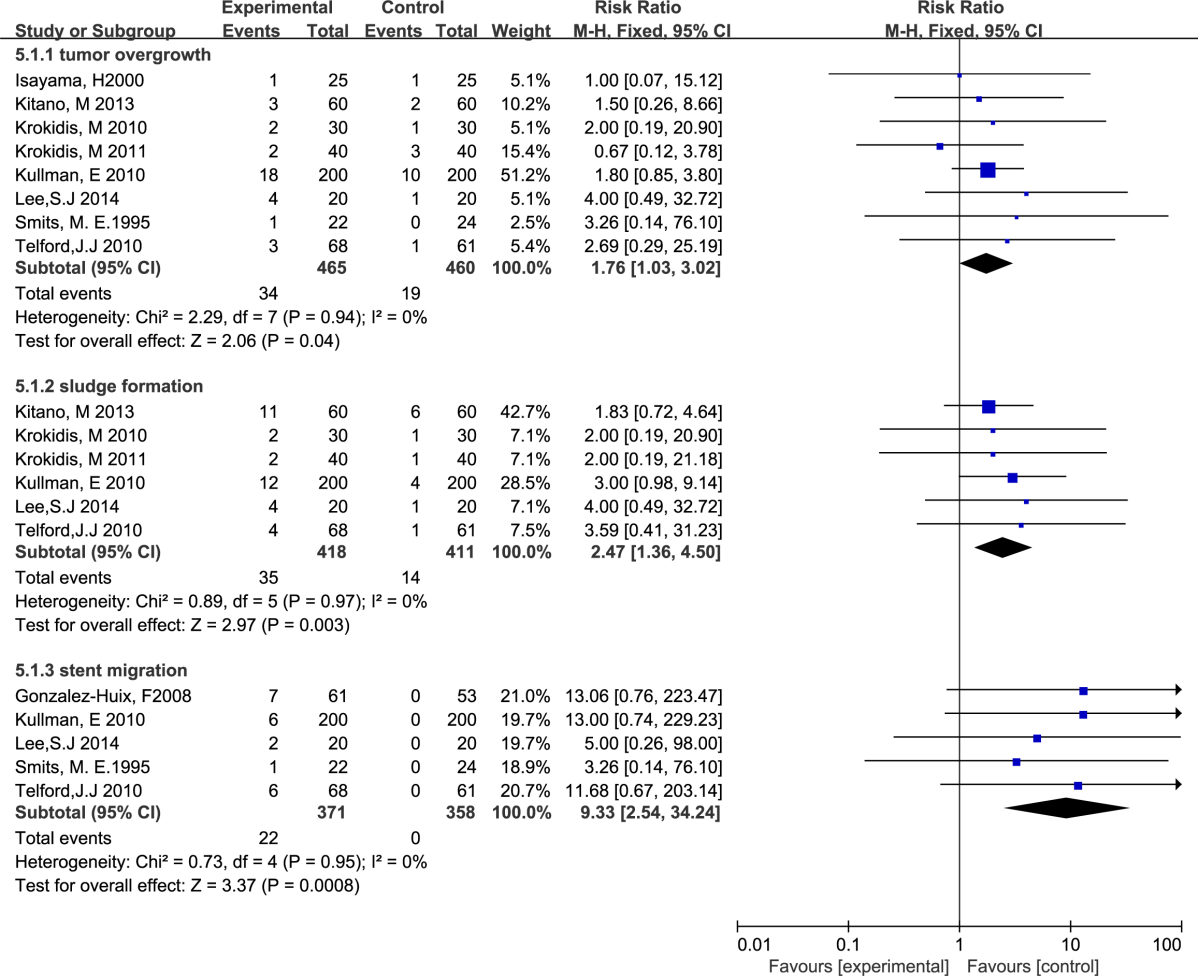
Forest plot of tumor overgrowth, sludge formation and stent migration.

Ten studies involving 1225 participants reported stent dysfunction affecting 154 patients with UCSEMSs and 142 patients with CSEMSs. For overall stent dysfunction, the studies showed no significant difference between the two groups, with significant between-study heterogeneity, as shown in Fig 4 (RR 0.85, M-H, random, 95% CI 0.57–1.25; p = 0.83, I^2^ = 63%). However, the rates of sludge formation (RR 2.47, M-H, fixed, 95% CI 1.36–4.50; p = 0.003, I^2^ = 0%), stent migration (RR 9.33, M-H, fixed, 95% CI 2.54–34.24; p = 0.008, I^2^ = 0%) and tumor overgrowth (RR 1.76, M-H, fixed, 95% CI 1.03–3.02; p = 0.04, I^2^ = 0%) were higher in the patients with CSEMSs compared with those with UCSEMSs (Figs 4 and 5). In contrast, the rate of tumor ingrowth in the UCSEMS group was significantly higher than the rate in the CSEMS group (RR 0.25, M-H, random, 95% CI 0.12–0.52; p = 0.002, I^2^ = 40%) (Fig 5).

#### Complication rate

**Fig 6:**
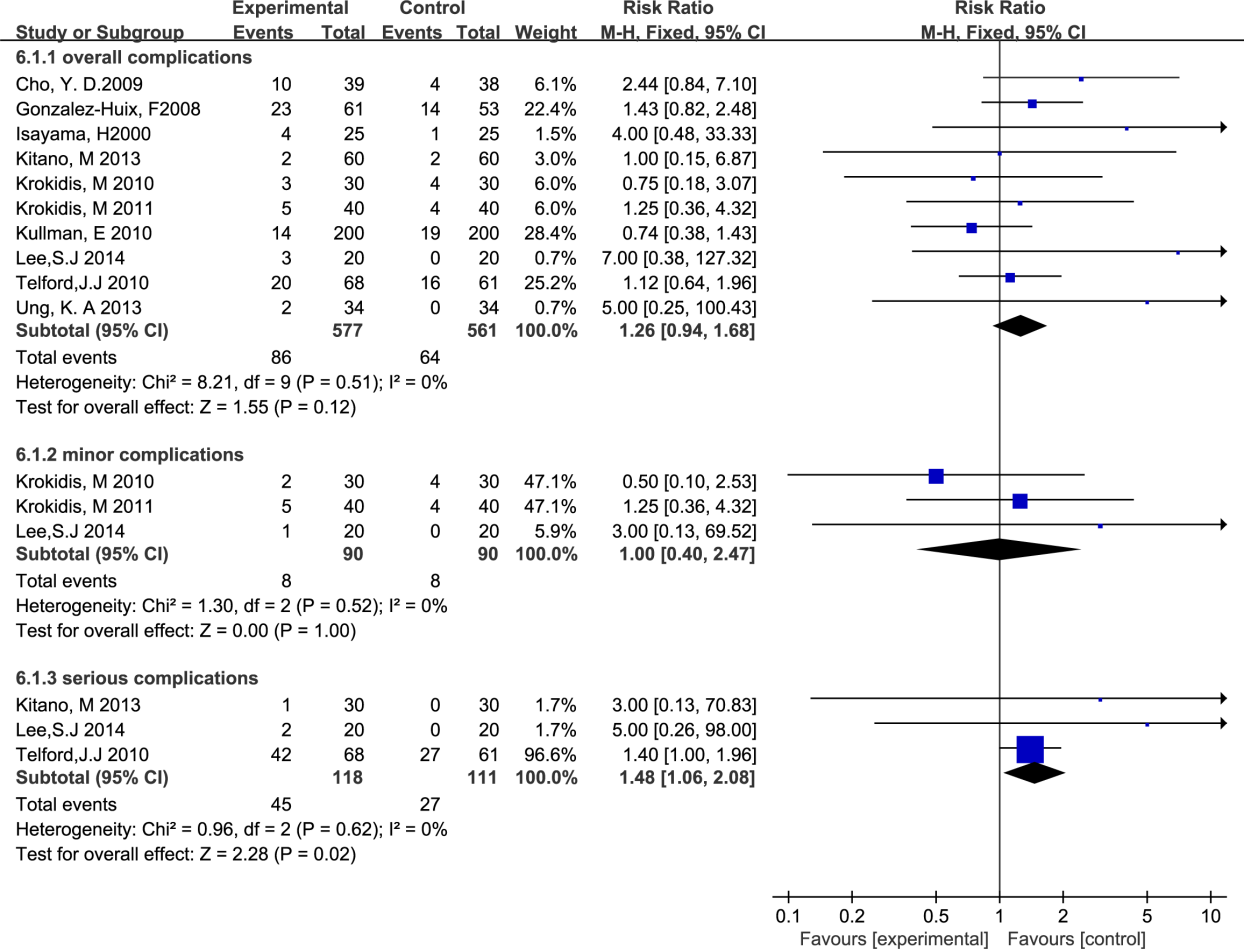
Forest plot of overall complications, minor complications and serious complications.

A summary of the studies (Fig 6) revealed no difference in the overall complication rate (RR 1.26, M-H, fixed, 95% CI 0.94–1.68; p = 0.12, I^2^ = 0%). A subgroup analysis did not indicate a significant difference in outcomes between the two groups regarding minor complication diagnoses (RR 1.00, M-H, fixed, 95% CI 0.40–2.47; p = 1.0, I^2^ = 0%). However, a higher rate of severe complications was suggested for CSEMSs compared with UCSEMSs (RR 1.48, M-H, fixed, 95% CI 1.06–2.08; p = 0.02, I^2^ = 0%).

#### Biliary drainage efficacy

**Fig 7:**
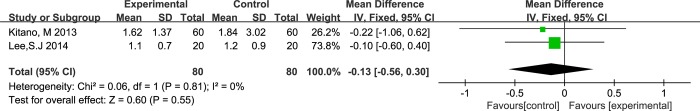
Forest plot of the change in bilirubin.

Changes in serum bilirubin after stent insertion were only reported in two studies [14, 25]. After the stent was inserted for two weeks, no significant differences were observed (Fig 7) between the two arms (MD-0.13, IV fixed, 95% CI -0.56–0.3; p = 0.55, I^2^ = 0%).

## Discussion

Patients with inoperable malignant distal biliary obstruction have poor quality of life and bad prognoses. The remarkable advantages of SEMS displacement include longer stent patency and fewer repeat insertions compared with plastic stents [33, 34]. Nonetheless, stent dysfunctions caused by sludge formation and tumor ingrowth are the main problems affecting the use of UCSEMSs, whereas CSEMSs were designed to avoid tumor ingrowth. However, whether CSEMSs or UCSEMSs provide better therapeutic efficacy remains unclear. Thus, we performed this review to investigate whether the efficacies of the two types of stents differ significantly.

Our meta-analysis included 14 eligible prospective RCTs that compared cumulative stent patency, overall patient survival, and complication rates between UCSEMSs and CSEMSs. Of the 14 trials, 7 were published as abstracts, and the risks of bias for these trials are unknown. The sensitivity analysis excluded the 7 abstracts and remained consistent with the pooled trials. The 3 RCTs [25, 28, 30] concluded that UCSEMSs exhibited a significant longer mean patency than CSEMSs did. An opposite conclusion on stent patency was reported by Kitano *et al* and Krokidis *et al* [14, 15]. Our meta-analysis revealed no significant difference between the two groups, which is consistent with the remaining studies. However, a different conclusion regarding stent patency was reached by two previous meta-analyses. One study concluded that when CSEMSs were applied, they provided significantly longer stent patency than UCSEMSs did [20] (WMD 60.56 days; 95% CI 25.96–95.17; p = 0.001; 3 studies; I^2^ = 0%). In contrast, the other study suggested that the two stents did not significantly differ in the proportions of stent patency at 6 (OR 1.82; 95% CI 0.63–5.25) and 12 (OR 1.25; 95% CI 0.65–2.39) months [21]. However, the WMD in stent patency favored the CSEMSs (67.9 days 95% CI 60.3–75.5), presumably because only two trial data points could be merged. WMDs or odds ratios (ORs) were applied in the two previous meta-analyses [20, 21] to assess the duration and proportion of stent patency. The data were reported as means, ranges, medians, interquartile ranges or first quartiles, which limited data consolidation and therefore prevented the use of a summary value. These limitations may be why the two meta-analyses failed to reach a unanimous conclusion. Moreover, stent patency and patient survival are time-to-event outcomes; notably, ORs only measure the number of events, which is less appropriate for analyzing time-to-event outcomes. The most appropriate analytical approach for time-to-event outcomes is the HR, which considers the number and timing of events [24]. Several studies [12–16, 25, 26] have reported that using cumulative stent patency is more suitable for comparing stent patency between two groups, and thus, we considered this measure in our meta-analysis. In particular, we extracted HRs from a Kaplan-Meier graph of cumulative stent patency. However, a summary of the HRs suggests no significant difference in cumulative stent patency (HR 0.93, 95% CI 0.19–4.53; p = 0.93), without significant between-study heterogeneity (Chi^2^ = 0.31, df = 6, I^2^ = 0%).

Patient survival mainly depends on the tumor type, metastatic rate and eventual comorbidities. We pooled the data from the selected studies and found no differences in the etiological factors and tumor metastatic rates between the two groups. Three studies [12, 16, 26] reported tumor metastases in 36% of patients in the UCSEMS group and 42% of patients in the CSEMS group. However, we did not address the eventual comorbidities in the two groups. In our meta-analysis, only one study [15] suggested a significant difference in patient survival between the UCSEMS and the CSEMS groups (median 180.5 vs 243.5; p = 0.039). In contrast, the remaining studies suggested no difference in overall survival between the two groups. Kitano *et al* [14] also examined the patient survival time without stent dysfunction, and they recorded a longer median time when CSEMSs were used compared with when UCSEMSs were used (median 187 vs 132 days; p = 0.043).

Although using SEMSs prolongs stent patency, stent dysfunction accounts for occlusion is the main problem associated with SEMSs. Stent dysfunction occurs for all types of SEMSs, but the mechanisms of different stent types differ. Sludge formation, tumor ingrowth, tumor overgrowth and stent migration are the main reasons for stent dysfunction. Although tumor ingrowth may occur in a covered stent segment because of covering membrane fissuring [35], several trials confirmed that UCSEMSs were more prone to biliary re-obstruction because of tumor ingrowth. Our review supports UCSEMSs exhibiting a higher rate of tumor ingrowth.

The SEMS coating membrane is intended to reduce recurrent biliary obstruction because of tumor ingrowth. Using covered stents has been shown to reduce the rate of tumor ingrowth, as stated above. However, our review shows that CSEMSs exhibit higher rates of sludge formation, tumor overgrowth and stent migration than UCSEMSs do. A previous study suggested that patients with CSEMSs show a higher rate of sludge formation than patients with UCSEMSs do (4–7% vs 0–3%) [14]. We speculated that the covering membrane favors microbial accumulation and thus is susceptible to bacterial biofilm development, as observed for plastic stents [5, 33]. Moreover, long stents with a high axial force kink easily, which slows biliary drainage and facilitates sludge formation. Because of the higher rate of migration observed in the CSEMS group, the coating membrane may prevent stent embedding, thus inhibiting stent integration into the bile duct wall. Moreover, stents with a high axial force can increase the risk of migration and bile duct kinking [36]. Interestingly, overall stent dysfunction did not differ significantly between CSEMSs and UCSEMSs. Two possible explanations for this observation are as follows. First, the benefits of preventing tumor ingrowth are offset by the adverse events caused by the covering membrane. Second, our meta-analysis only included the four main reasons (sludge formation, tumor ingrowth, tumor overgrowth and stent migration) for overall stent dysfunction; other reasons, such as food debris, were ignored because of incomplete data, although we attempted to contact the study authors about these data. Meta-regression was performed to explore the sources of heterogeneity existing in overall stent dysfunction and tumor ingrowth outcomes. However, data were presented in various formats in different studies, and incomplete data limited data consolidation. In this meta-analysis, the stent placement approach, the number of endoscopic sphincterotomy the research center and the region were regarded as variables in the meta-regression. Unfortunately, the four variables did not seem to contribute to the heterogeneity in the two outcomes cited above (S3 and S4 Figs). Rather, the heterogeneity might have resulted from the other clinical conditions, such as the stent length and stent diameter.

As stated above, our meta-analysis indicates that there is no significant difference in primary stent patency, overall stent dysfunction between two groups. Thus, there is a misunderstanding that the efficiency of the two stents for managing malignant distal biliary obstruction is equivalent. However, the fact is that the follow-up period in these RCTs only lasted from primary stents insertion to stents dysfunction or patients death, but not taken the further management for patients with occluded stent into account. The intervals and the rate of overall stent dysfunction were similar between two groups which also mean the ratio of patients who need further management is similar too. Kida M, *et al* [37]reported that nearly 50% survival patients with SEMSs had a stent occlusion, and the patency duration of further management with a balloon mechanical cleaning or “stent-in-stent” placement was rather short. Besides, removal of the occluded stents is better for preserving patient quality of life [37]. However, removal of UCSEMSs is more risky and tough than removal of CSEMSs because of tumor ingrowth [38, 39]. Familiari P, *et al* [40] reported that 0% of UCSEMSs and 86.4% of CSEMSs could be removed without serious complications after stents occlusion. A subsequent study [41] suggested that 38.4% occluded stent of UCSEMs was able to be removed, but the ratio was far lower than 92.3% of CSEMSs. Under this condition, in order to simplify the further management and preserve patient quality of life, CSEMSs is a better choice for patients with malignant biliary obstruction due to their removability.

Several investigators have reported that pancreatitis is the most common adverse event after SEMS placement, with an incidence range of 0–8.8% [34, 42–45]. Mechanical injuries from pancreatic duct manipulation and pancreatic orifice occlusion are the two major etiological causes of post-SEMS-placement pancreatitis. Moreover, SEMSs with a high axial force may increase the pancreatitis incidence compared with plastic stents. According to previous studies [1, 25, 46–48], CSEMSs might increase the incidence of pancreatitis and cholecystitis by covering the pancreatic duct orifice or cystic duct. We collected data on this topic from all but four of the selected trials [13, 27, 28, 31]; the excluded trials did not provide exact numbers. We found that of 464 patients with UCSEMSs, 24 suffered from cholecystitis, and 6 suffered from pancreatitis. Of the 472 patients who had CSEMSs, 25 suffered from cholecystitis, and 9 suffered from pancreatitis. Thus, we cannot conclude that patients with CSEMSs are prone to pancreatitis and cholecystitis. Moreover, none of the trials reported the pancreatic duct conditions or whether the pancreatic exocrine function remained normal. A distal bile duct that is obstructed or infiltrated by a tumor before SEMS placement may explain why CSEMSs near the papilla of Vater do not necessarily result in acute pancreatitis [16]. We also analyzed the overall complication rate, unexpectedly; CSEMSs did not reduce the overall complication rate after stent insertion compared with UCSEMSs. Additionally, we analyzed the complication classification between the two groups. The trials [15, 16, 25] classified the complications from minor to serious, in accordance with the Society of Interventional Radiology classification system for complications. Telford *et al* [13] defined a serious complication as one requiring an invasive procedure or hospitalization or resulting in death. The means used to grade the complications in the remaining studies were unclear. Interestingly, the overall complication, minor complication and serious complication rates did not differ between the two groups in only four trials [12, 14–16]. However, when we considered an additional trial [13], CSEMSs exhibited a higher rate of serious complications compared with UCSEMSs. Therefore, because the definition of a serious complication was inconsistent, different conclusions were reported.

Regarding biliary drainage efficiency, changes in serum bilirubin can be observed after stent insertion. Hyperbilirubinemia is a type of liver disorder that can produce biliary cirrhosis if the obstruction cannot be resolved for a long period of time. Many patients cannot bear the pruritus caused by high-level bilirubin. For patients undergoing palliative treatment, biliary drainage plays a fundamental role in improving quality of life. To assess the biliary drainage efficacy, we compared the changes in serum bilirubin at 2 weeks after stent insertion between the two groups. Only two studies [14, 25] reported serum bilirubin values, and our review suggested no significant difference between UCSEMSs and CSEMSs. The change in serum bilirubin at 2 weeks after stent placement can be used as a short-term evaluation of biliary drainage. In the present analysis, the success rate of stent deployment did not vary, which is the main reason for the equivalent biliary drainage between the two groups, and the biliary drainage did not differ.

Certain limitations of our meta-analysis should be noted. First, although we included many studies, we did not investigate the potential publication bias of the abstracts included in this review. Second, although the patient characteristics (age and gender) did not differ, important information, such as eventual comorbidities, stent length, axial force and the stent placement approach, could not be pooled, which might have affected the conclusions regarding clinical efficacy.

## Conclusions

Our meta-analysis indicates that patients with malignant distal biliary obstructions treated with CSEMSs do not experience an extra benefit compared with patients treated with UCSEMSs during the period from primary stent insertion to primary stent dysfunction or patient death. However, given the further management for occluded stent, we believe that using CSEMSs is a better choice for patient undergoing palliative treatment for malignant distal biliary obstruction.

## Supporting Information

S1 PRISMA Checklist PRISMA checklist. (DOC)

S1 Fig Each risk-of-bias item presented as a percentage across all included studies. (TIF)

S2 Fig Each risk-of-bias item for each included study. (TIF)

S3 Fig Meta-regression for the overall stent dysfunction outcome. (JPG)

S4 Fig Meta-regression for the tumor ingrowth outcome. (JPG)

S1 Table Search strategy. (DOC)
