# Supplementary figures and images for: Covered versus Uncovered Self-Expandable Metal Stents for Managing Malignant Distal Biliary Obstruction: A Meta-Analysis
Source: PLoS One. 2016 Feb 9;11(2):e0149066. doi: 10.1371/journal.pone.0149066 (PMC4747571; doi:10.1371/journal.pone.0149066)

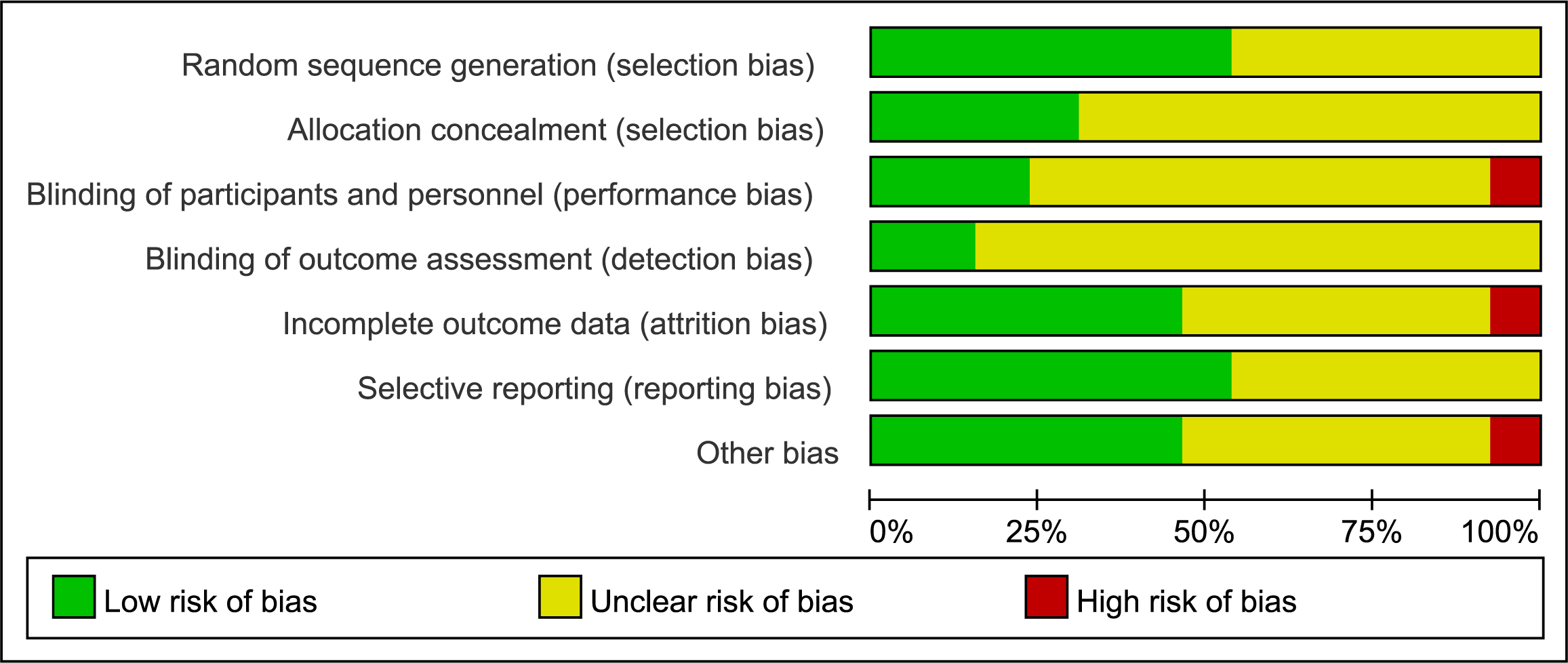

Supplement: S1 Fig — (TIF) [file pone.0149066.s002.tif]

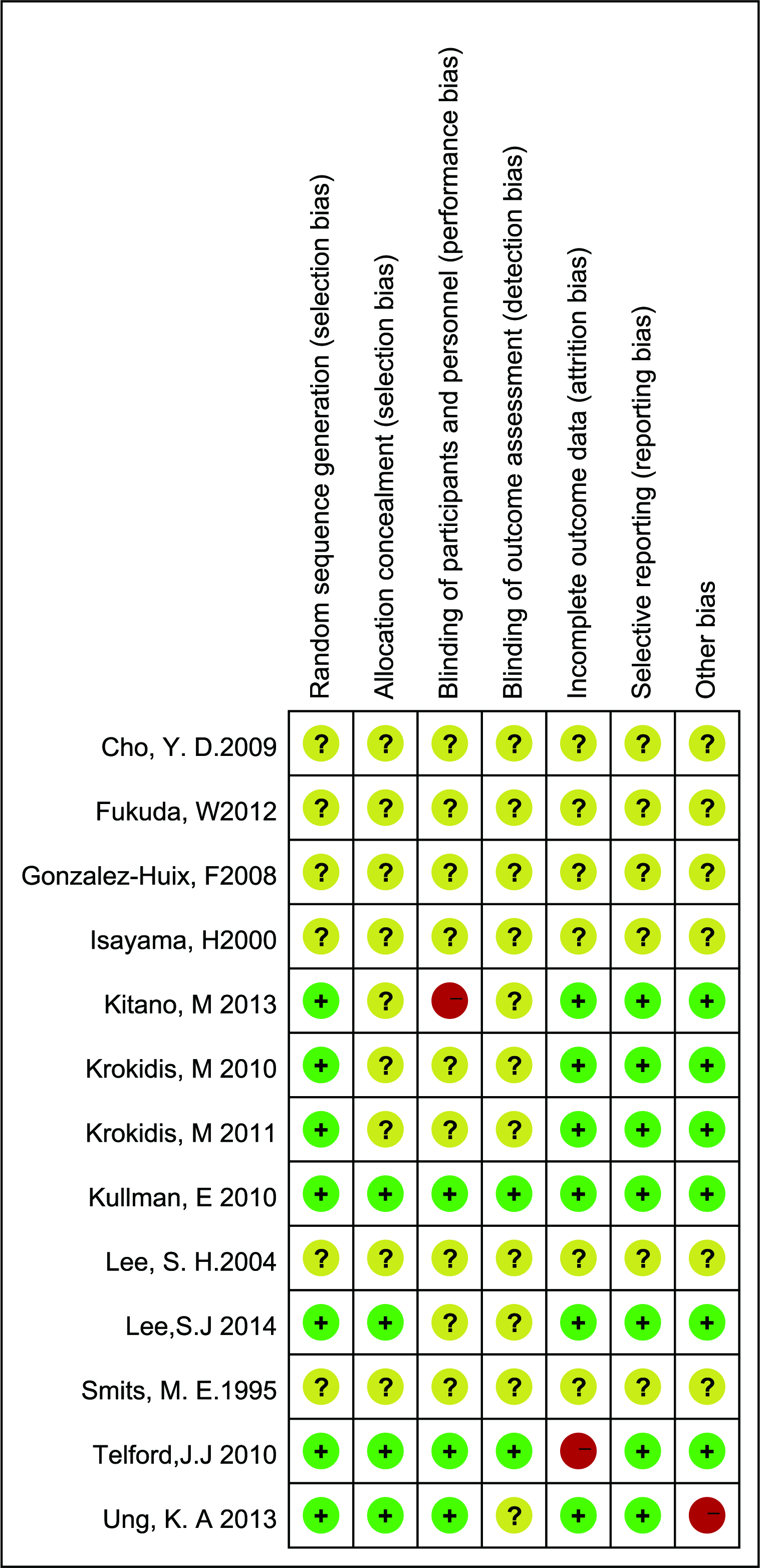

Supplement: S2 Fig — (TIF) [file pone.0149066.s003.tif]

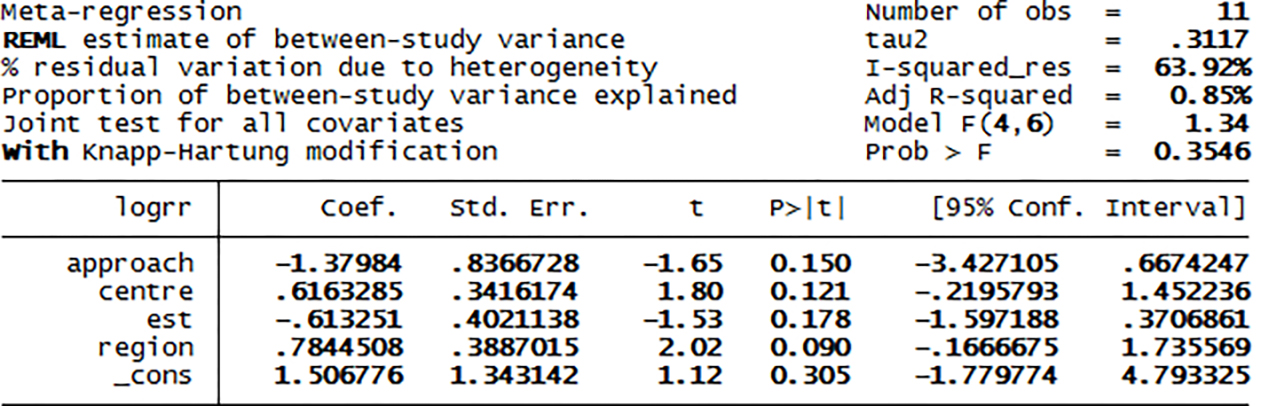

Supplement: S3 Fig — (JPG) [file pone.0149066.s004.jpg]

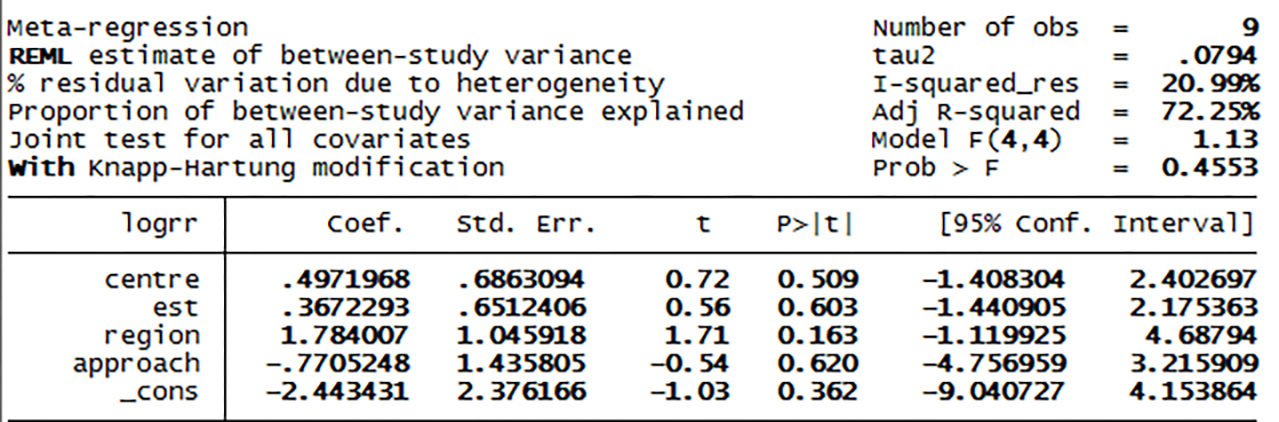

Supplement: S4 Fig — (JPG) [file pone.0149066.s005.jpg]
