# Supplementary material for: Covered versus Uncovered Self-Expandable Metal Stents for Managing Malignant Distal Biliary Obstruction: A Meta-Analysis
Source: PLoS One. 2016 Feb 9;11(2):e0149066. doi: 10.1371/journal.pone.0149066 (PMC4747571; doi:10.1371/journal.pone.0149066)
Supplement: S1 Table — (DOC) [file pone.0149066.s006.doc]

**S1 Table.** Search strategy

| **Cochrane Library databases,** from 1980 to May 20, 2015 |
| --- |
| #1 MeSH descriptor: [Pancreatic Neoplasms] explode all trees  #2 (((((neoplas*) or cancer*) or carcin*) or tumo*) or metasta*) or malig*  #3 pancrea*  #4 #2 and #3  #5 MeSH descriptor: [Common Bile Duct Neoplasms] explode all trees  #6 ((bile) and duct) and ((((((neoplas*) or cancer*) or carcin*) or tumo*) or metasta*) or malig*)  #7 MeSH descriptor: [Bile Duct Neoplasms] explode all trees  #8 MeSH descriptor: [Cholestasis] explode all trees  #9 ((bile) and duct) and obstruct*  #10 cholestas*  #11 MeSH descriptor: [Common Bile Duct Diseases] explode all trees  #12 MeSH descriptor: [Jaundice, Obstructive] explode all trees  #13 (obstruct*) and jaundice*  #14 ((malig*) and bil*) and obstruct*  #15 (bil*) and strictur*  #16 #1 or #4 or #5 or #6 or #7 or #8 or #9 or #10 or #11 or #12 or #13 or #14 or #15  #17 MeSH descriptor: [Stents] explode all trees  #18 (stent*) or endoprosthesis  #19 #17 or #18  #20 #16 and #19 Publication Year from1980 to 2015 |
| **MEDLINE (PubMed),** from 1980 to May 20, 2015 |
| #1 Search "Pancreatic Neoplasms"[Mesh]  #2 Search (((((neoplas*) OR cancer*) OR carcin*) OR tumo*) OR metasta*) OR malig*  #3 Search pancrea*  #4 Search (pancrea*) AND ((((((neoplas*) OR cancer*) OR carcin*) OR tumo*) OR metasta*) OR malig*)  #5 Search "Common Bile Duct Neoplasms"[Mesh]  #6 Search ((bile) AND duct) AND ((((((neoplas*) OR cancer*) OR carcin*) OR tumo*) OR metasta*) OR malig*)  #7 Search "Bile Duct Neoplasms"[Mesh]  #8 Search "Cholestasis"[Mesh]  #9 Search ((bile) AND duct) AND obstruct*  #10 Search cholestas*  #11 Search "Common Bile Duct Diseases"[Mesh]  #12 Search "Jaundice, Obstructive"[Mesh]  #13 Search (obstruct*) AND jaundice*  #14 Search ((malig*) AND bil*) AND obstruct*  #15 Search (bil*) AND strictur*  #16 Search (((((((((((("Pancreatic Neoplasms"[Mesh]) OR ((pancrea*) AND ((((((neoplas*) OR cancer*) OR carcin*) OR tumo*) OR metasta*) OR malig*))) OR "Common Bile Duct Neoplasms"[Mesh]) OR (((bile) AND duct) AND ((((((neoplas*) OR cancer*) OR carcin*) OR tumo*) OR metasta*) OR malig*))) OR "Bile Duct Neoplasms"[Mesh]) OR "Cholestasis"[Mesh]) OR (((bile) AND duct) AND obstruct*)) OR cholestas*) OR "Common Bile Duct Diseases"[Mesh]) OR "Jaundice, Obstructive"[Mesh]) OR ((obstruct*) AND jaundice*)) OR (((malig*) AND bil*) AND obstruct*)) OR ((bil*) AND strictur*)  #17 Search "Stents"[Mesh]  #18 Search (stent*) OR endoprosthesis  #19 Search (((stent*) OR endoprosthesis)) OR "Stents"[Mesh]  #20 Search (((((randomized controlled trial[pt]) OR (controlled clinical trial[pt]) OR (randomized[tiab]) OR (placebo[tiab]) OR (drug therapy[sh]) OR (randomly[tiab]) OR (trial[tiab]) OR (groups[tiab])) NOT (animals[mh] NOT humans[mh]))))  #21 Search ((((((((((((((("Pancreatic Neoplasms"[Mesh]) OR ((pancrea*) AND ((((((neoplas*) OR cancer*) OR carcin*) OR tumo*) OR metasta*) OR malig*))) OR "Common Bile Duct Neoplasms"[Mesh]) OR (((bile) AND duct) AND ((((((neoplas*) OR cancer*) OR carcin*) OR tumo*) OR metasta*) OR malig*))) OR "Bile Duct Neoplasms"[Mesh]) OR "Cholestasis"[Mesh]) OR (((bile) AND duct) AND obstruct*)) OR cholestas*) OR "Common Bile Duct Diseases"[Mesh]) OR "Jaundice, Obstructive"[Mesh]) OR ((obstruct*) AND jaundice*)) OR (((malig*) AND bil*) AND obstruct*)) OR ((bil*) AND strictur*))) AND ((((stent*) OR endoprosthesis)) OR "Stents"[Mesh])) AND ((((((randomized controlled trial[pt]) OR (controlled clinical trial[pt]) OR (randomized[tiab]) OR (placebo[tiab]) OR (drug therapy[sh]) OR (randomly[tiab]) OR (trial[tiab]) OR (groups[tiab])) NOT (animals[mh] NOT humans[mh]))))) Publication Year from1980 to 2015 |
| **EMBASE,** from 1980 to May 20, 2015 |
| #1 Pancreatic Neoplasms.mp. or exp pancreas tumor/  #2 (neoplas* or cancer* or carcin* or tumo* or metasta* or malig*).mp.  #3 pancrea*.mp. or exp pancreas/  #4 2 and 3  #5 Common Bile Duct Neoplasms.mp. or exp bile duct tumor/  #6 ((bile and duct and (neoplas* or cancer* or carcin* or tumo* or metasta* or malig*)).mp.  #7 Bile Duct Neoplasms.mp. or exp bile duct tumor/  #8 exp cholestasis/ or Cholestasis.mp.  #9 (bile and duct and obstruct*).mp.  #10 Common Bile Duct Diseases.mp. or exp common bile duct disease/  #11 Jaundice, Obstructive.mp. or exp obstructive jaundice/  #12 (malig* and bil* and obstruct).mp.  #13 (bil* and strictur*).mp.  #14 1 or 4 or 5 or 6 or 7 or 8 or 9 or 10 or 11 or 12 or 13  #15 Stents.mp. or exp stent/  #16 exp endoprosthesis/ or endoprosthesis.mp.  #17 15 or 16  #18 (random* or blind* or placebo* or meta-analys*).mp.  #19 14 and 17 and 18   #20 limit 19 to yr="1980-Current" |
| **Science Citation Index Expanded,** from 1980 to May 20, 2015 |
| #1 ((pancrea*) AND ((((((neoplas*) OR cancer*) OR carcin*) OR tumo*) OR metasta*) OR malig*))  #2 (((bile) AND duct) AND ((((((neoplas*) OR cancer*) OR carcin*) OR tumo*) OR metasta*)  OR malig*))  #3 (Common Bile Duct Neoplasm*)  #4 (Cholestas*)  #5 (Common Bile Duct Disease*)  #6 ((obstruct*) AND jaundice*)  #7 (((malig*) AND bil*) AND obstruct*)  #8 ((bil*) AND strictur*)  #9 (bile AND duct AND obstruct*)  #10 #9 OR #8 OR #7 OR #6 OR #5 OR #4 OR #3 OR #2 OR #1  #11 (stent* OR endoprosthesis)  #12 (random* or blind* or placebo* or meta-analys*)  #13 #12 AND #11 AND #10 Publication Year from1980 to 2015 |
